# Uncovering the hidden complexity of multicellular magnetotactic bacteria

**DOI:** 10.1371/journal.pbio.3002695

**Published:** 2024-07-12

**Authors:** Kayla S. Stoy, William C. Ratcliff

**Affiliations:** School of Biology, Georgia Institute of Technology, Atlanta, Georgia, United States of America

## Abstract

Multicellular magnetotactic bacteria (MMB) have a surprisingly complex multicellular lifestyle. This Primer explores a new study in PLOS Biology which combines genomics, microscopy, and isotopic labeling to show that MMB form obligately multicellular consortia of genetically diverse cells with rudimentary division of labor.

## Teaser

Multicellular magnetotactic bacteria (MMB) have a surprisingly complex multicellular lifestyle. A new study in *PLOS Biology* combines genomics, microscopy, and isotopic labeling to show that MMB form obligately multicellular consortia of genetically diverse cells with rudimentary division of labor.

The evolution of multicellularity was transformative for life on Earth, fueling biological innovation and underpinning the origin of novel ecosystems. Bacteria pioneered this major evolutionary transition, first evolving multicellular bodies over 3 billion years ago [1]. Despite this long history, bacterial multicellularity is often overlooked, as bacteria rarely form the large, long-lived, obligately multicellular bodies we generally associate with multicellular life. However, in recent years, microbiologists have begun to reveal the rich social lives of multicellular bacteria, from the wolf-pack hunting behavior of myxobacteria [2] to bacterial collectives that have evolved adaptive immunity against viruses [3]. In this issue of *PLOS Biology*, Schaible and colleagues [4] use a suite of cutting-edge techniques to provide an unprecedented look into the hidden complexity of a fascinating multicellular bacterial group—multicellular magnetotactic bacteria (MMB).

MMB are most often found in marine sediments with vertical chemical gradients. These bacteria possess the remarkable ability to orient themselves along the Earth’s geomagnetic field lines, which in combination with chemotaxis and aerotaxis, allows them to navigate to their preferred position within these chemical gradients [5,6]. This behavior is made possible by the presence of specialized organelles called magnetosomes. These lipid vesicles contain either magnetite (Fe_3_O_4_) or greigite (Fe_3_S_4_) crystals, which are biomineralized within the organelles that are arranged in chains along the cytoskeleton of each MMB cell. This creates a magnetic dipole within each cell that passively aligns it with the Earth’s geomagnetic field. Importantly, the alignment of these magnetosome chains is consistent across the multiple cells that make up an MMB consortium. It is this coordinated alignment of magnetic dipoles across all the cells that enables the entire multicellular MMB consortium to efficiently orient and navigate as a unit [7]. Researchers like Schaible and colleagues also leverage this unique feature and use magnetic enrichment to isolate these otherwise uncultivable organisms from sediment samples.

In this paper, Schaible and colleagues show that, in addition to their incredible range of magnetotactic, aerotactic, phototactic, and chemotactic behaviors [8], MMB are unique in another aspect. They are the first example of an organism that is both obligately multicellular, lacking a unicellular stage capable of independent growth, and that is also composed of genetically diverse cells (as opposed to every cell being essentially identical). While some questions remain about the life cycle of MMB, prior work has shown that when MMB cells are removed from their consortia, they die, suggesting that these organisms do not have a persistent single-cell life history stage [9]. To uncover the multicellular nature of MMB, Schaible and colleagues employed a range of cutting-edge culture-independent techniques.

**Fig 1:**
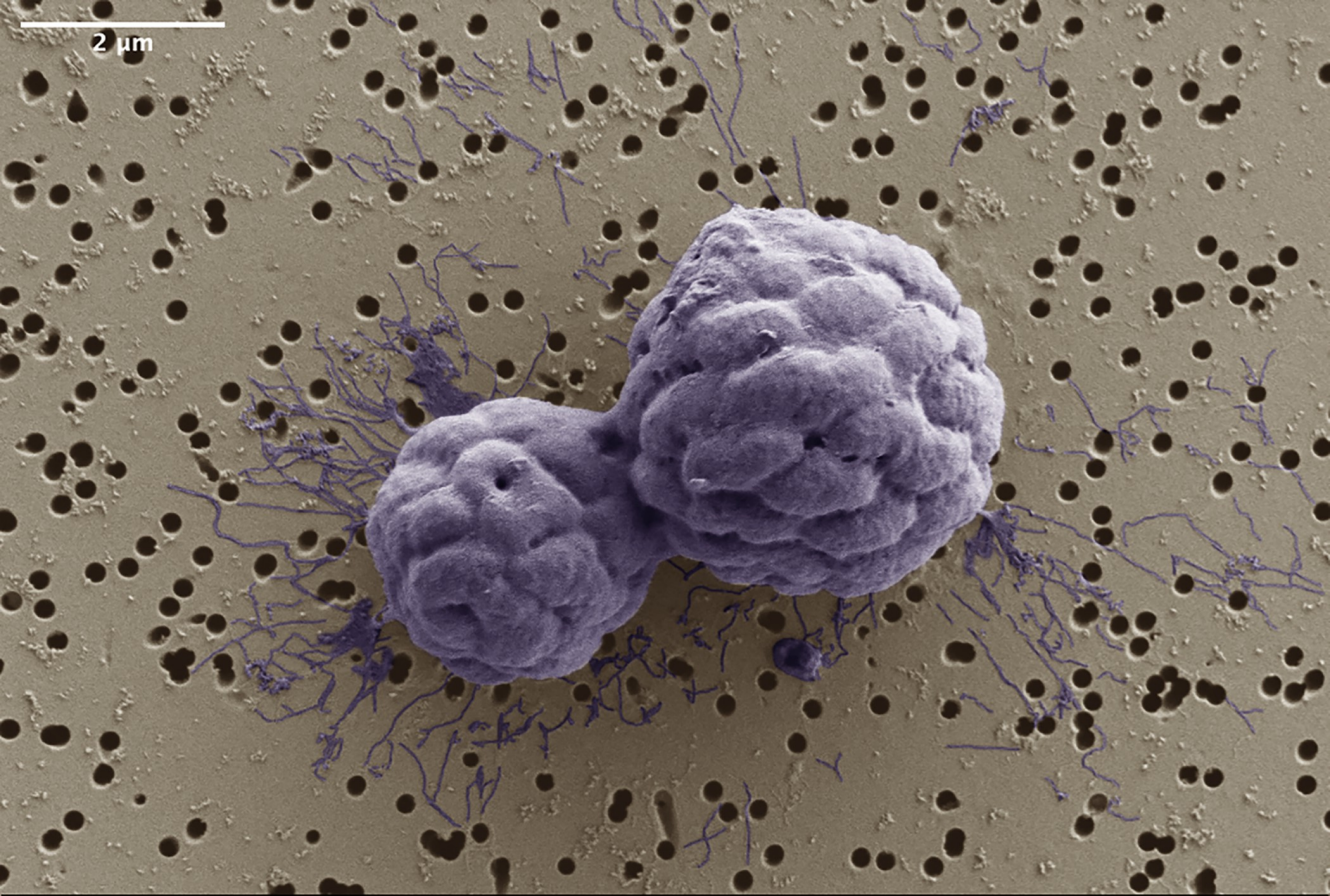
Multicellular magnetotactic bacterial consortia possibly caught in the act of division. Image courtesy of George Schaible, Montana State University.

Using microscopy, the team observes MMB consortia that appear to reproduce by dividing along an acellular region at their center, producing two multicellular daughters (Fig 1). Given that MMB grow via cells “staying together” after division, one would expect a consortium to be clonal, yet this is not the case. Using metagenomics, Schaible and colleagues uncover a stunning amount of genetic diversity within MMB consortia—up to 100× more diversity than was found within clonal multicellular controls! This suggests that the genetic variation within MMB consortia cannot be explained by *de novo* mutation, but instead must arise from another mechanism, such as between-group cellular exchange.

Cells within MMB also exhibit metabolic heterogenaity. The authors examined MMB consortia with Nanoscale Secondary Ion Mass Spectrometry (NanoSIMs), a powerful technique for quantifying fine-scale cellular metabolic activity. Within a single MMB consortium, the researchers observed “hot spots” where specific cells metabolized isotopically labeled carbon substrates as well as heavy water at increased rates relative to other cells in the same consortium. The authors then demonstrate that protein synthesis also varies across the consortium, concentrated near the acellular central region, using Bioorthogonal Noncanonical Amino Acid Tagging (BONCAT), an approach in which cells uptake noncanonical amino acids that are used during protein synthesis and are later detected via fluorescence microscopy [10].

What does this metabolic heterogeneity mean? The authors speculate that MMB cells may be metabolically differentiated. For example, certain cells may specialize in the metabolism of specific substrates, like acetate, sharing the resulting resources with other cells via the central acellular space. This type of metabolic specialization is a hallmark of a division of labor, where different cells perform distinct tasks required for overall organismal function. While the presence of metabolic heterogeneity suggests the possibility of division of labor in MMB, direct evidence of full-fledged division of labor in these organisms remains to be demonstrated. Nonetheless, this paper provides tantalizing hints that MMB may have achieved a remarkable degree of functional integration.

The findings of Schaible and colleagues open up exciting new avenues for exploring bacterial multicellularity. The observation of non-clonal MMB consortia raises the question of how genomic heterogeneity is generated and maintained during MMB replication. What does the complete multicellular life cycle look like? Do groups fuse to one another, and if so, when and how? Being able to bring these organisms into the lab and grow them in stable monoculture may be an important next step, as it would rule out a hidden unicellular phase. Future work should also fully explore the intracellular division of labor—what molecules are being exchanged, and is this dependent on the genetic diversity of cellular constituents?

Perhaps the most important implication of this work is that it expands our conceptual understanding of the potential diversity of basic multicellular life cycles. Until now, we have generally assumed that obligately multicellular organisms would be clonal, and all prior examples support this assumption [11]. This is important: because all other obligately multicellular organisms are also clonal, it has proven difficult to disentangle the role of each of these traits on the evolution of multicellularity. Prior research suggests that both obligate multicellularity and clonality may independently play a role in facilitating multicellular adaptation [11,12], and novel model systems that decouple these traits may prove instrumental in testing these hypotheses.

Overall, recent work has shown that bacterial multicellularity is fundamentally different than what we have come to expect from large, long-lived, functionally integrated multicellular eukaryotes. From the synchronized swimming of magnetotactic bacteria to the coordinated predation of myxobacteria, it is clear that bacteria have evolved a wide range of social behaviors uniquely suited to their specific ecologies. As we continue to explore the frontiers of bacterial multicellularity with an ever-expanding toolkit, we are likely to uncover even more surprises that will challenge our assumptions about the nature and potential of multicellular life on Earth.
